# Orbital blowout fractures: manifestations and missed diagnoses in 207 surgically treated patients

**DOI:** 10.4317/medoral.26559

**Published:** 2024-04-14

**Authors:** Matti Nikunen, Rayan Nikkilä, Johanna Snäll

**Affiliations:** 1 MD, DDS. Department of Oral and Maxillofacial Diseases, University of Helsinki and Helsinki University Hospital, Helsinki, Finland; 2 DDS, PhD. Research Program in Systems Oncology, Faculty of Medicine, University of Helsinki, Helsinki, Finland; 3 MD, DDS, PhD. Department of Oral and Maxillofacial Diseases, University of Helsinki and Helsinki University Hospital, Helsinki, Finland

## Abstract

**Background:**

We aimed to retrospectively measure the incidence of missed orbital blowout fracture diagnosis in primary examinations of patients with surgically treated fractures, to identify the causes of the diagnostic oversight, and to describe the clinical manifestations of the fractures.

**Material and Methods:**

A retrospective cohort of all patients with unilateral orbital blowout fractures who underwent subsequent surgical fracture reduction at the Department of Oral and Maxillofacial Diseases, Helsinki University Hospital, from January 2011 to July 2021, was gathered. Demographics, fracture type, associated injuries, clinical manifestations, reconstruction indication, diagnostic delay, and causes of fractures were analysed. For statistical analysis, Fisher’s exact test, unpaired t-test, and the Wilcoxon Rank Sum were used. Significance was set at P˂0.05.

**Results:**

Fracture diagnosis was missed in 26 (13%) of 207 patients: in 40% of patients aged under 18 years and in 10% of patients aged 18 years or over (*P*=0.005). Suboptimal eye examination was found in 62% of patients with missed fracture and in 13% of those with timely diagnosis (*P*<0.001). Adjusted odds ratios for missed diagnosis in patients aged under 18 years versus patients aged 18 years and over was 9.3 (95% CI 2.4-35) and in patients with suboptimal versus sufficient eye examination 13.6 (95% CI 5.1-37). More common clinical manifestations in patients aged under 18 years were diplopia or restricted eye movements (*P*=0.005), pain in eye movements (*P*=0.010), nausea and/or vomiting (*P*<0.001), and bradycardia (*P*=0.014); periorbital haematoma was rarer (*P*<0.001). Suboptimal eye examination was involved in 62% and misinterpretation of computed tomography images in 50% of missed fractures, together explaining 85% of cases.

**Conclusions:**

Orbital blowout fractures are often missed in primary examination, especially in children and adolescents, who also present with subtler clinical manifestations. While the diagnosis can be difficult, appropriate clinical and radiological examination will reveal most cases.

** Key words:**Orbital blowout fracture, diagnostic delay, paediatric fracture, surgical treatment, signs and symptoms, oculocardiac reflex.

## Introduction

Orbital blowout fractures involve fracturing of the thin orbital floor or medial wall bones, with the orbital rim remaining intact (1). These fractures typically arise from a blunt hydraulic or buckling mechanism, or a combination of both (2).

Delayed treatment leads to prolonged suffering for the patient and may have severe consequences. These include vision loss due to retrobulbar haematoma compressing the optic nerve, worsened ocular motility or persistent diplopia due to muscle incarceration and ischaemia, and oculocardiac reflex due to vagal stimulation with potentially life-threatening sequelae (3-9). Prompt diagnosis and intervention can be critical for minimising the long-term impact of orbital blowout fractures (6-8,10-12).

Symptoms of orbital blowout fractures range from mild to severe and include impaired ocular motility, diplopia, globe malposition, vagal symptoms, emphysema, pain, swelling, ecchymosis, and paresthesia (13-16). Children typically sustain a trapdoor type fracture with less obvious outward signs of trauma than adults (8,14,16,17). While paediatric fractures pose a particular diagnostic dilemma (18), challenges remain in all age groups(19).

We aimed to retrospectively assess the occurrence of missed orbital fractures in primary evaluation, to identify the causes behind the diagnostic oversight, and to describe the clinical manifestations of orbital blowout fractures in patients with subsequently reconstructed orbital blowout fractures. We hypothesise that missing the diagnosis in primary evaluation is more common in patients under 18 years of age.

Materal and Methods

- Study design

A retrospective cohort study was designed to evaluate diagnostic accuracy in surgically treated unilateral orbital blowout fracture patients. Data from all patients with orbital floor and/or medial wall fractures requiring reconstructive surgery at the Department of Oral and Maxillofacial Diseases, Helsinki University Hospital, from 1 January 2011 to 31 July 2021, were collected.

- Inclusion and exclusion criteria

Patients requiring surgery for unilateral orbital blowout fracture (i.e. fracture of the orbital floor and/or medial wall without other facial fractures extending to the orbit) due to blunt trauma were included in the study. Excluded were patients with fractures extending to the orbital rim, patients with bilateral fractures requiring reconstruction, and patients who did not seek medical evaluation within 21 days of the initial injury.

- Study variables

The outcome variable was missed diagnosis of an isolated orbital fracture by a health care professional. A missed diagnosis was established when a fracture was not suspected or diagnosed during the patient’s primary evaluation at the primary health care visit.

To evaluate the significance of patients´ age in missing the diagnosis, the primary predictor variable was age grouped as children (˂18 years) or adults (≥18 years). Additionally, age groups were stratified into the following subgroups: 1) <12 years, 2) 12-17 years, 3) 18-64 years, and 4) ≥65 years.

Additional predictor variables were clinical symptoms and findings, which were categorised as periorbital haematoma, diplopia and/or restricted eye movement, subconjunctival haemorrhage, infraorbital nerve injury, pain in eye movements or opening, soft tissue entrapment/impingement (confirmed during surgery), swelling impeding clinical examination, facial wound requiring suturing, nausea and/or vomiting, eye injury (requiring ophthalmological treatment and/or follow-up), and bradycardia (defined as heart rate ˂50 in the acute setting for adults and ˂60 for children aged under 12 years).

Explanatory variables were sex, injury mechanism, suboptimal evaluation of eye status, orbital blowout fracture type, presence of combined facial fractures not extending to the orbit, associated injuries outside the facial region, and loss of consciousness (confirmed by an eyewitness) and/or radiological intracranial injury. Injury mechanisms were grouped into the following seven categories: 1) assault, 2) ground-level fall, 3) sports or recreational accident, 4) fall from height, and 5) traffic accident. Suboptimal evaluation of eye status was determined to have occurred when eye movements, double vision, or subjective visual acuity was not recorded. Orbital blowout fractures were classified as orbital 1) floor fracture, 2) medial wall fracture, or 3) combined fracture of floor and medial walls.

Associations of explanatory and predictor variables, as well as clinical manifestations and age groups, with missed diagnosis were evaluated. Number of days from primary evaluation to fracture diagnosis and its causes were reported. In addition, indications for surgery (clinical symptom or orbital volume growth) were presented.

- Statistical analyses

The association between categorical variables was tested using Fisher’s exact test. Means and medians were compared using the unpaired t-test and the Wilcoxon Rank Sum test, respectively. Statistical significance was set at P˂0.05. Binary logistic regression was employed to calculate adjusted odds ratios for age and suboptimal eye evaluation. All statistical analyses were performed using R software (The R Project for Statistical Computing), version 4.3.1.

## Results

**Figure 1:**
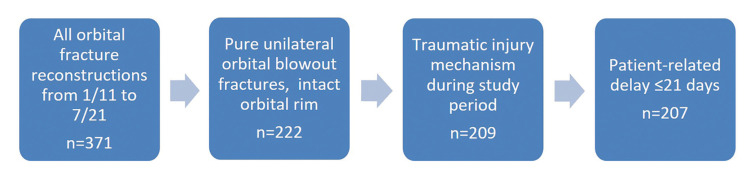
Data collection process.

Data collection is described in detail in Fig. 1. A total of 207 patients with reconstructed orbital fractures were included. All fracture diagnoses were confirmed radiologically by computed tomography (CT) imaging.

The mean age of all 207 patients was 44 years (range 5.4-91, median 41). Associations between explanatory variables and age groups are described in Table 1. There were more males in both age groups, 87% in children and 62% in adults. Injury mechanisms differed between the groups (*P*<0.001). Sports accidents (53%) were the most common cause in children, whereas assaults (45%) were the most common cause in adults. Fractures were more extensive in adults, with 38% being combined fractures of the orbital floor and medial wall, compared with 6.7% in children (*P*=0.001).

In 26 (13%) of 207 patients, the diagnosis was missed. Table 2 compares explanatory variables of patients with and without missed diagnosis. Suboptimal eye examination was found in 16 (62%) of 26 patients with missed diagnosis (*P*<0.001). In patients with timely diagnosis, 23 (13%) of 181 eye examinations were defined as suboptimal (*P*<0.001). Injury mechanisms were different between patients aged under 18 years and patients aged 18 years and over (*P*<0.001). Sports and recreational injuries were the predominant cause of injury in patients aged under 18 years, accounting for 67% (10 of 15) of cases. In patients aged 18 years and over, the leading cause was assault, comprising 45% (87 of 192) of cases. Injury mechanism was associated with missed diagnosis (*P*=0.046). Orbital floor fractures were more likely to get missed than combined or medial wall fractures (*P*<0.001).

A missed fracture diagnosis occurred in 40% of children and 10% of adults (*P*=0.005). In patients aged 12-18 years and under 12 years, 22% and 67% of diagnoses, respectively, were initially missed (Table 3) (*P*=0.003). Adjusted odds ratio for missed diagnosis in patients under 18 years versus patients 18 years and over was 9.3 (95% CI 2.4-35) and in patients with suboptimal versus sufficient eye examination 13.6 (95% CI 5.1-37).

**Figure 2:**
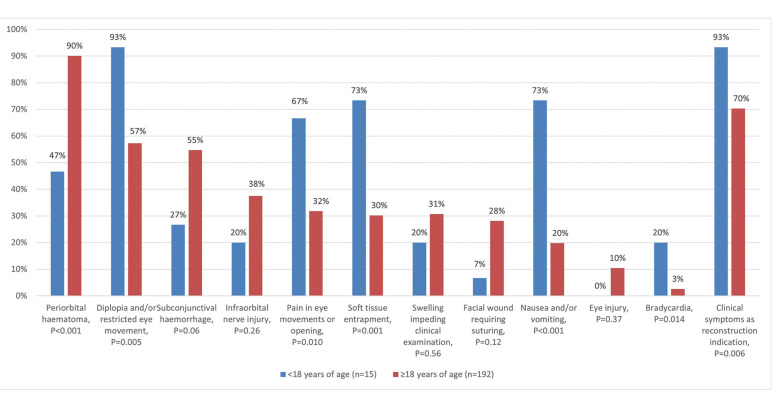
Manifestations of orbital blowout fractures in patients aged under 18 years and in patients aged 18 years and over.

Children’s blowout fractures exhibited distinct clinical manifestations relative to adults. The most common manifestations included diplopia and/or restricted eye movements (93% vs. 57%, *P*=0.005), pain in eye movements or opening (67% vs. 32%, *P*=0.010), soft tissue entrapment (73% vs. 30%, *P*=0.001), nausea and/or vomiting (73% vs. 20%, *P*<0.001), and bradycardia (20% vs. 2.6%, *P*=0.014). Periorbital haematoma was rarer in children than in adults (47% vs. 90%, *P*<0.001) (Fig. 2).

Clinical symptom (restricted eye movements, diplopia, or significant GMP) was the reconstruction indication in 93% and 70% of cases among children and adults, respectively (*P*=0.006) (Fig. 2). No differences were observed in the reconstruction indication when comparing patients with missed and timely diagnoses (Fig. 2).

**Figure 3:**
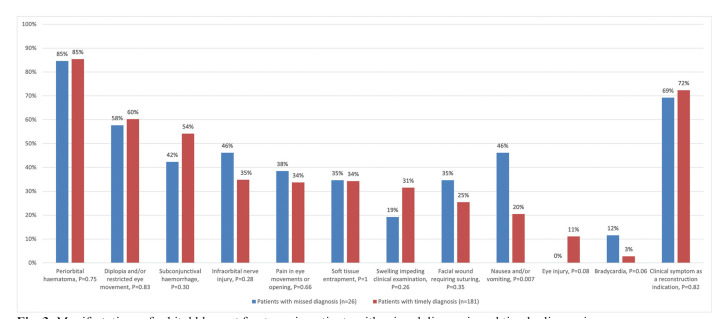
Manifestations of orbital blowout fractures in patients with missed diagnosis and timely diagnosis.

Patients with missed diagnosis presented a clinical picture similar to that of patients with timely diagnosis (Fig. 3). Nausea and/or vomiting was more common in patients with missed diagnoses: 12 of 26 (46%) vs. 37 of 181 (20%) (*P*=0.007). There were no eye injuries in patients with missed diagnoses.

The causes for missing a fracture diagnosis were identified and classified into three partially overlapping categories. Suboptimal eye examination was determined in 16 patients (62%) and misinterpretation of imaging studies in 13 patients (50%), 6 of whom concurrently had suboptimal eye examinations. In 10 patients (38%), imaging studies were not ordered. Suboptimal examination and misinterpretation of imaging studies together explained 22 (85%) of the 26 cases (Table 4).

No significant differences were noted in associated injuries, combined facial fractures, or radiological brain injuries between patients with missed and timely diagnoses or between age groups.

## Discussion

Diagnosis of orbital blowout fractures can be demanding and failure to accurately diagnose this condition can lead to a worse outcome in eye functions and aesthetics and, while rare, can be potentially life-threatening due to the oculocardiac reflex (5,8,9,11,12). Our purpose was to describe the occurrence of primarily missed orbital fracture diagnosis and identify contributing factors to the diagnostic oversights as well as to describe the clinical manifestations of these fractures. We hypothesised that a higher incidence of missed diagnoses occurs in patients under 18 years of age.

The hypothesis was confirmed, with children and adolescents being nine times more likely to have their fractures missed in primary evaluation. Up to 40% of patients aged under 18 years had a missed fracture. In turn, in patients aged 18 years or over misses occurred in 10%. The diagnostic challenges were emphasised in the youngest age group; in patients under 12 years, 67% of diagnoses were missed (Table 3). Suboptimal eye status was the main cause (OR 13.6) and further evaluation showed also challenges in assessing the need for radiological examination and interpreting CT images.

In general, the rates and definitions of missed injuries in the literature vary, with rates ranging from 1.1% to as high as 65%. Higher rates are often related to orthopaedic extremity injuries and blunt injury mechanisms (20-22). Paediatric fractures are often more subtle and show unique fracture patterns compared with adults (23,24). This is also reflected in clinical manifestations. Apparent outward signs, such as periorbital haematoma and subconjunctival haemorrhage, were less common in children than in adults (Fig. 2). However, children were more symptomatic than older subjects. Diplopia and/or restricted eye movement, pain in eye movements or opening, nausea and/or vomiting, and bradycardia were all significantly more common among children than among adults (Fig. 2). This was evident in reconstruction indications, as 93% of children underwent reconstruction due to acute symptomatic presentation. In contrast, 30% of adults were asymptomatic, and orbital volume growth was the primary indication of reconstruction. Injury mechanisms were different between age groups, with sports and recreational injuries being more common in children and interpersonal violence in adults (*P*<0.001), as reported in previous literature (25).

Bradycardia was recorded in 8 (3.9%) out of 207 patients and was more common in children (*P*=0.01) (Fig. 2), 3 (20%) of 15 presenting with bradycardia. Oculocardiac reflex can be defined as a 20% lowering of heart rate due to traction of extraocular muscles or globe pressure, and other classic symptoms are a drop of blood pressure, nausea and syncope (26). The retrospective study design limited the analysis to heart rate limits of 60 and 50 in the acute setting for patients aged 18 years and over and patients aged under 18 years, respectively. A possible oculocardiac symptom of nausea or vomiting was present in 24% of all orbital fracture patients. This was emphasised in those under 18 years of age, with 73% presenting with nausea or vomiting. Many of these patients’ symptoms indicated oculocardiac origin, yet this was not recognised in the clinical setting. Under-recognition of oculocardiac reflex has been noted in previous reports (27), but the retrospective nature of this study did not allow for definitive identification of all oculocardiac reflexes. Nausea can complicate cooperation and examination and can be confused with nausea related to alcohol or drug abuse, but orbital injury as a cause should be kept in mind. No sequelae were related to bradycardia in this patient cohort.

Suboptimal evaluation of eye status was observed in 62% of those with missed diagnosis (Table 4). Misinterpretation of imaging studies was also common, found in 50% of cases with initially missed fracture, followed by lack of imaging studies at first presentation (38%). Our findings highlight the importance of comprehensive eye status, the need for additional imaging studies in midfacial injuries, and the need for on-call radiologists and clinicians to be able to interpret CT images of the orbital area. In 6 out of 13 cases with misinterpretation of imaging studies, only a head CT was obtained, resulting in the fracture being visible only at the lower edge of the scanned area, hindering detection of intracranial injuries. While 28% of orbital fractures were reconstructed due to a large volume change in CT and not due to a clinical symptom (i.e. restricted eye movements, diplopia, or significant GMP), in careful tertiary hospital evaluation all subjects displayed some clinical manifestations resulting from the injury.

Although 80% of patients under 18 years had a comprehensive eye examination as a part of their primary health care examination, 40% of their fractures were missed. Ocular motility was evaluated with the subject following a moving finger or pen with their gaze. The “finger test” is not very sensitive in detecting restricted eye movements relative to a full orthoptic examination (28), yet in tertiary hospital examination diplopia or restricted eye movements were detected in 93% of these patients. This emphasizes the importance of diligent examination in order to identify ocular manifestations and the underlying fracture.

The retrospective nature of the study and the reliance on documentation constitute study limitations. Recording of clinical manifestations may be poor, and patient cooperation can be incomplete. Patients do not always notice or report all orbital blowout fracture symptoms immediately after injury, and, for example, emphysema may develop days later. In this study, we included symptoms both at primary health care evaluation and at the tertiary hospital, where often a more thorough examination is performed. The sample size, particularly the number of paediatric patients, is also small. We only included patients who underwent surgical fracture treatment; unoperated blowout fracture patients do not appear in the data.

Clinically relevant orbital blowout fractures were often missed in primary health care examination, especially in children and adolescents. Although children often display milder outward signs of orbital fracture, they tend to be more symptomatic, presenting frequently with limited eye functions, pain, nausea, and bradycardia. These constitute important clues for the clinician, as a careful examination of imaging studies and eye function in facial injuries, coupled with an understanding of the subtler symptoms associated with orbital fractures, can enhance diagnostic accuracy and guide the selection of appropriate imaging modalities across all age groups.

## Figures and Tables

**Table 1:** Associations between study variables and age group in 207 patients with orbital blowout fracture.

Study variables		Patients under 18 year of age		Patients of 18 years or more		*p*
		n		% of n	n	% of n
All		15	7	192	93	-
Sex	Male	13	10	119	90	0.09
	Female	2	3	73	97	
Injury mechanism	Assault	2	2	87	98	<0.001
	Ground-level fall	2	3	58	97	
	Sports or recreational accident	10	29	25	71	
	Fall from height	1	8	11	92	
	Traffic accident	0	0	11	100	
Suboptimal eye examination	No	12	7	156	93	1.0
	Yes	3	8	36	92	
Orbital blowout fracture type	Floor	12	9	118	91	0.001
	Combined	1	1	73	99	
	Medial wall	2	67	1	33	
Combined facial fracture (not extending to the orbit)	No	14	9	141	91	0.12
	Yes	1	2	51	98	
Associated injuries	No	14	9	138	91	.12
	Yes	1	2	54	98	
Loss of consciousness and/or radiological intracranial injury	No	13	8	153	92	0.70
	Yes	2	5	39	95	

**Table 2:** Associations between study variables and missed orbital blowout fracture diagnosis in 207 patients with orbital blowout fracture.

Study variables		Patiens with missed diagnosis		Patiens with timely diagnosis		*p*
		n		% of n	n	% of n
All		26	13	181	87	-
Sex	Male	18	14	114	86	0.66
	Female	8	11	67	89	
Injury mechanism	Assault	6	7	83	93	0.046
	Ground-level fall	7	12	53	88	
	Sports or recreational accident	9	26	26	74	
	Fall from height	2	17	10	83	
	Traffic accident	2	18	9	82	
Suboptimal eye examination	No	10	6	158	94	<0.001
	Yes	16	41	23	59	
Orbital blowout fracture type	Floor	17	13	114	87	0.41
	Combined	1	2	65	98	
	Medial wall	8	80	2	20	
Combined facial fracture not extending to the orbit	No	20	13	135	87	1.0
	Yes	6	12	46	88	
Associated injuries	No	21	14	131	86	0.48
	Yes	5	9	50	91	
Loss of consciousness and/or radiological intracranial injury	No	21	13	145	87	0.76
	Yes	5	12	36	88	
Diagnostic delay (days)	Mean	6.2	-	0.1	-	0.01
	Median	2.5		0		<.001
	Range	0-60		0-5	

**Table 3:** Associations between patients' age and missed orbital blowout fracture diagnosis in 207 patients with orbital fracture.

Patients' age		Patiens with missed diagnosis		Patiens with timely diagnosis		*p*
		n		% of n	n	% of n
All		26	13	181	87	-
Age groups (years)	< 18	6	40	9	60	0.005
	≥ 18	20	10	172	90	
Age subgroups (years)	< 12	4	67	2	33	0.003
	12-17	2	22	7	78	
	18-64	15	10	139	90	
	≥ 65	5	13	33	87	

**Table 4:** Description of causes for missed orbital blowout fracture diagnosis in 26 patients.

Causes for missed diagnosis	Patiens with missed diagnosis	
	n	%
Suboptimal eye examination	16	62
No eye examination	6	23
Misinterpretation of imaging studies	13	50
Head CT only	6	23
No imaging studies at first presentation	10	38
Sufficient eye examination, no imaging	4	15

## References

[1] Patterson RW Jr, Depue RV Jr (1962). Blow-out fracture of the orbit. Am J Ophthalmol.

[2] Kono S, Vaidya A, Takahashi Y (2023). Mechanisms of Development of Orbital Fractures: A Review. Ophthalmic Plast Reconstr Surg.

[3] Dubois L, Steenen SA, Gooris PJ, Mourits MP, Becking AG (2015). Controversies in orbital reconstruction--II. Timing of post-traumatic orbital reconstruction: a systematic review. Int J Oral Maxillofac Surg.

[4] Jeffrey J, Nelson F, Hohlbein J, Mehta A, Davies B (2022). South Texas orbital fracture protocol for emergency department evaluation of orbital fractures. Am J Emerg Med.

[5] Byeon JY, Choi HJ (2017). Orbital Cellulitis Following Orbital Blow-out Fracture. J Craniofac Surg.

[6] Damgaard OE, Larsen CG, Felding UA, Toft PB, von Buchwald C (2016). Surgical Timing of the Orbital "Blowout" Fracture: A Systematic Review and Meta-analysis. Otolaryngol Head Neck Surg.

[7] Yamanaka Y, Watanabe A, Rajak SN, Nakayama T, Sotozono C (2022). Correlation between surgical timing and postoperative ocular motility in orbital blowout fractures. Graefes Arch Clin Exp Ophthalmol.

[8] Grant JH 3rd, Patrinely JR, Weiss AH, Kierney PC, Gruss JS (2002). Trapdoor fracture of the orbit in a pediatric population. Plast Reconstr Surg.

[9] Mallinson FB, Coombes SK (1960). A hazard of anaesthesia in ophthalmic surgery. Lancet.

[10] Egbert JE, May K, Kersten RC, Kulwin DR (2000). Pediatric orbital floor fracture : direct extraocular muscle involvement. Ophthalmology.

[11] Gudnadottir G, Hammarfjord O, Johansson S, Hellgren J (2023). Pediatric Orbital Fractures: Outcomes in Relation to Time of Surgery. J Craniofac Surg.

[12] Harris GJ (2006). Orbital blow-out fractures: surgical timing and technique. Eye (Lond).

[13] Ray CN, Marsh HD, Gilmore JE, Kirk DW, Larumbe-Zabala E, Freedman KA (2023). Review of 451 Patients Presenting With Orbital Wall Fractures: A Retrospective Analysis. J Craniofac Surg.

[14] Hammond D, Grew N, Khan Z (2013). The white-eyed blowout fracture in the child: beware of distractions. J Surg Case Rep.

[15] Eom T, Kim Y (2015). Analysis of symptoms according to areas of orbital floor in orbital inferior wall fractures. J Craniofac Surg.

[16] Buck LS, Stockton S, Spankovich C, Jordan JR (2020). Pediatric orbital floor fractures and the oculocardiac reflex: Experience from a level I trauma center. American Journal of Otolaryngology.

[17] Chi MJ, Ku M, Shin KH, Baek S (2010). An analysis of 733 surgically treated blowout fractures. OphthalmologicaJournal international d'ophtalmologieInternational journal of ophthalmologyZeitschrift fur Augenheilkunde.

[18] Collin J, Afshar F, Thomas S (2015). Medial Wall Fracture and Orbital Emphysema Mimicking Inferior Rectus Entrapment in a Child. Craniomaxillofac Trauma Reconstr.

[19] Kumar S, Artymowicz A, Muscente J, Shinder R, Mostafavi D (2023). Do Not Fall for This; Diagnostic Challenges in Orbital Floor Fractures With Extraocular Muscle Entrapment. Cureus.

[20] Wilbers A, DeHoet CA, Sliter RJ, Noland A, Quinn KR, Lightwine K (2022). An analysis of missed injuries at a level 1 trauma center with a tertiary survey protocol. Am J Surg.

[21] Janjua KJ, Sugrue M, Deane SA (1998). Prospective evaluation of early missed injuries and the role of tertiary trauma survey. J Trauma.

[22] Enderson BL, Reath DB, Meadors J, Dallas W, DeBoo JM, Maull KI (1990). The Tertiary Trauma Survey: A Prospective Study of Missed Injury. Journal of Trauma and Acute Care Surgery.

[23] George MP, Bixby S (2019). Frequently Missed Fractures in Pediatric Trauma: A Pictorial Review of Plain Film Radiography. Radiol Clin North Am.

[24] Alcala-Galiano A, Arribas-Garcia IJ, Martin-Perez MA, Romance A, Montalvo-Moreno JJ, Juncos JM (2008). Pediatric facial fractures: children are not just small adults. Radiographics.

[25] Bera RN, Tiwari P, Pandey V (2022). Does Early Treatment of Paediatric Orbital Fracture Offer Any Advantage in Terms of Post-Operative Clinical Outcomes. J Maxillofac Oral Surg.

[26] Meuwly C, Chowdhury T, Sandu N, Golanov E, Erne P, Rosemann T (2017). Definition and Diagnosis of the Trigeminocardiac Reflex: A Grounded Theory Approach for an Update. Front Neurol.

[27] Mehmood N, Hasan A (2021). Oculocardiac Reflex: An Underrecognized But Important Association With Orbital Trap Door Fractures. Pediatr Emerg Care.

[28] Timkovic J, Stransky J, Handlos P, Janosek J, Tomaskova H, Stembirek J (2021). Detecting Binocular Diplopia in Orbital Floor Blowout Fractures: Superiority of the Orthoptic Approach. Medicina (Kaunas).

